# Radiomics features on radiotherapy treatment planning CT can predict patient survival in locally advanced rectal cancer patients

**DOI:** 10.1038/s41598-019-51629-4

**Published:** 2019-10-25

**Authors:** Jiazhou Wang, Lijun Shen, Haoyu Zhong, Zhen Zhou, Panpan Hu, Jiayu Gan, Ruiyan Luo, Weigang Hu, Zhen Zhang

**Affiliations:** 1 Department of Radiation Oncology, Fudan University Shanghai Cancer Center, Shanghai, 200032 China; 2 Department of Oncology, Shanghai Medical College, Fudan University, Shanghai, 200032 China; 3 Perelman Center for Advanced Medicine, Philadelphia, PA 19104 USA; 4 MAASTRO Clinic, Maastricht, Netherlands

**Keywords:** Cancer imaging, Tumour biomarkers

## Abstract

This retrospective study was to investigate whether radiomics feature come from radiotherapy treatment planning CT can predict prognosis in locally advanced rectal cancer patients treated with neoadjuvant chemoradiation followed by surgery. Four-hundred-eleven locally advanced rectal cancer patients which were treated with neoadjuvant chemoradiation enrolled in this study. All patients’ radiotherapy treatment planning CTs were collected. Tumor was delineated on these CTs by physicians. An in-house radiomics software was used to calculate 271 radiomics features. The results of test-retest and contour-recontour studies were used to filter stable radiomics (Spearman correlation coefficient > 0.7). Twenty-one radiomics features were final enrolled. The performance of prediction model with the radiomics or clinical features were calculated. The clinical outcomes include local control, distant control, disease-free survival (DFS) and overall survival (OS). Model performance C-index was evaluated by C-index. Patients are divided into two groups by cluster results. The results of chi-square test revealed that the radiomics feature cluster is independent of clinical features. Patients have significant differences in OS (p = 0.032, log rank test) for these two groups. By supervised modeling, radiomics features can improve the prediction power of OS from 0.672 [0.617 0.728] with clinical features only to 0.730 [0.658 0.801]. In conclusion, the radiomics features from radiotherapy CT can potentially predict OS for locally advanced rectal cancer patients with neoadjuvant chemoradiation treatment.

## Introduction

The current standard treatment for locally advanced (T3-4 and/or N+) rectal cancer is neoadjuvant chemoradiation followed by total mesorectal excision (TME) surgery. Given the potential morbidity associated with the standard approach, there is growing interesting in risk-stratifying patients to identify those who may safely forgo TME without sacrificing disease control^1^. Recent research focuses on optimization of neoadjuvant treatment strategies, including integrating neoadjuvant chemotherapy, the non-surgical ‘watch and wait’ approach for patients with complete response or de-intensified adjuvant chemotherapy for patients with tumor downstaging. Thus, it is essential to predict and stratify patients for the application this treatment response-based adaptive strategy. However, in current clinical practice, the response evaluation, such as digital rectal examination, endoscopic assessment and image modalities (MR/PET-CT), have been investigated, but good correlation has not been demonstrated^2,3^.

In addition, numerous studies have been conducted to determine the prognostic factors for locally advanced rectal cancer after neoadjuvant chemoradiation. Most of these studies are based on traditional clinical characteristics, such as ypTNM staging^4^, tumor regression grade (TRG)^5,6^, pathological complete response (pCR) rate^7^ and CEA level^8,9^. By combining these prediction factors, effective c-indices (concordance indices) for external validation (local control, 0.68; distant control, 0.73; overall survival, 0.70) were achieved^9^. This performance is clinically useful but is still not optimal. The addition of other knowledge domains to the prediction model, such as image feature analysis, is expected to increase model accuracy.

Novel ‘omic’ research, such as genomics, is investigated in some studies^10^. Radiomics is an innovative image feature analysis that extract data from medical images acquired from daily clinical practice. With the exception of anatomical information, there are many classes of image features, including texture features, wavelet features and fractal features, in medical images^11^. The link between image features and tumor prognosis has been demonstrated by numerous researchers. Radiomics is an emerging field that extracts advanced features from non-invasive images to quantitatively describe tumor phenotypes using automatic algorithms^12^. Compared with genomics and proteomics, radiomics has the advantages of non-invasion, a more comprehensive view of tumor and convenience in routine practice; thus, this technique has great potential for use in individualized treatment. Recent studies have reported its potential clinical applications in the prediction of prognosis^11,13^, response assessment^14,15^ and tumor staging^16,17^. However, many factors can affect final radiomics models, for example, the image acquisition machine and parameters, image pre-processing algorithm, image segmentation and the modeling method. All of these factors have associated uncertainties that can affect the quality of the final radiomics model^18^.

To date, relatively few studies with small number of patients have focused on radiomics in the response of neoadjuvant chemoradiation and prognosis in locally advanced rectal. And most of those studies was using MRI images^19^ and FDG PET images^20^. CT image can be used to predict lymph node metastasis in colorectal cancer^21^. It is unknown whether radiomics features on radiotherapy treatment planning CT can predict patient surivival in locally advanced rectal cancer patients.

Therefore, the aim of our study is to investigate whether radiomics features on radiotherapy treatment planning CT can predict the outcome of locally advanced rectal cancer patients who were receive with neoadjuvant chemoradiation therapy; and to establish a prediction model between radiomics and outcome.

## Methods

### Study design and patients

**Figure 1:**
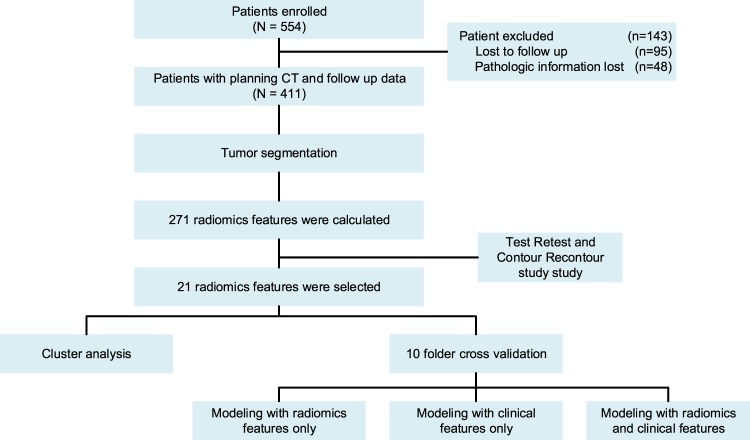
The workflow of this study.

This retrospective study was approved by the Fudan University Shanghai Cancer Center Institutional Review Board and all methods were performed in accordance with the guidelines and regulations of this ethics board and the Hospital Ethics Committee agreed to the informed consent waiver. From 2007 to 2015, a cohort of 554 consecutive patients with locally advanced (cT3-4 and/or cN1-2) rectal cancer treated with neoadjuvant chemoradiotherapy followed by surgery at the Fudan University Shanghai Cancer Center was identified from the colorectal cancer database. Among these patients, 95 patients were excluded due to missing information, and 411 patients were enrolled into analysis and modeling. These patients’ planning CTs were collected. All CT images were not contrast-enhanced. The voxel size was 1.12 mm (0.98–1.20). We use 128 discretization when calculating 2^nd^ order radiomics feature. No addition preprocessing was performed. All images were imported into MIM (MIM Software Inc. Cleveland, OH) and then contoured by two physicians. One physician is a radiologist who specialized in rectal imaging with 5 years of experience and another is a radiation oncologist who specialized in gastrointestinal cancer with 3 years of experience. An in-house radiomics software was used to calculate 271 radiomics features. The details of the feature calculation algorithm are based on a previous study^22^, and the item of the radiomics features were provided in Supplementary Table S1. Based on the results of test-retest and contour-recontour, 21 radiomics features were selected. Two statistical methods were implemented to get a reliable result, including cluster analysis and cross validation-based multivariable modeling. The performance of prediction model with the radiomics or clinical features were calculated. The outcomes we focused on in this study include local control, distant control, disease free survival and overall survival. The workflow of this study is presented in Fig. 1.

### Test-retest and contour-recontour

The test-retest and contour recontour were imperative to obtain reliable radiomics results. Briefly, for the test-retest study, 40 rectal cancer patients with stage II were included retrospectively in this study. All patients underwent two baseline clinical CT scans within an average 8.7 days (5 to 17 days) at Fudan University Shanghai Cancer Center before any treatment was delivered. Both scans were obtained with the same CT scanner using the same imaging protocol (350 mA tube current, 120 kVp tube voltage, 0.92 × 0.92 mm pixel size, 5-mm thickness, 512 × 512 matrix). These patients’ medical images were divided into two groups: scan 1 and scan 2. For test-retest task, the rectal tumor was distinguished and segmented by a radiation oncologist. Spearman’s correction coefficients were calculated for each radiomics features. Features with correction coefficient > 0.7 and correction coefficient with volume < 0.8 were selected. The details of this study are reported study^23^.

For the contour-recontour study, 31 local advanced rectum patients were used. The radiotherapy planning CT, which was acquired before treatment, was collected. The parameters of the CT scanner were same as the test-retest study. For contour-recontour task, the tumor was segmented by one radiation oncologist and one radiologist. Spearman’s correction coefficients were calculated for each radiomics features. Features with a correction coefficient > 0.7 and correction coefficient with a volume < 0.8 were selected.

### Modeling and statistical method

To obtain reliable results and avoid over fitting, we use two modeling and statistical methods to analyze our data, including an unsupervised method and a supervised method. All modeling and statistical calculations were performed in R (http://CRAN.R-project.org/).

For the unsupervised methods, non-negative matrix factorization (NMF)-based cluster was implemented^24^. To determine how many groups were needed for this dataset, we applied non-negative matrix factorization (NMF) with different group numbers and randomly repeated the method 20 times to evaluate the stability of this group number. Then, the optimal group number was used to cluster patients. After patient clustering, a chi-square test was used to investigate the relation between clinical features and radiomics-based clustering.

For the supervised method, a 10-fold cross-validation-based multivariable modeling strategy was implemented to fit final model. Briefly, the entire dataset was randomly partitioned into 10 groups of roughly equal size. All samples except the first subset (90% patients, approximately 370 patients) were used as a training dataset. The selected samples (10% patient, approximately 41 patients) were predicted by this model and used to estimate performance measures. The first subset returned to the training set, and procedures were repeated with the second selected subset held out, etc. For the model training, first features with auto-correlation > 0.95 were filtered by the CARET package of R^25^. Missing values were imputed using the MASS package of R. Then, features with a p-value < 0.05 (Log-rank test for discrete variable, cox model for continuous variable) were selected, and a backward stepwise method was implemented with AIC = 1. The c-index was calculated for the training and testing datasets. C-index = 0.5 implies no predictive ability (no better than random guessing), and c-index = 1 implies a perfect prediction ability. These calculations were performed by the RMS package of R^26^.

### Ethics approval and consent to participate

This study was approved by the Institutional Review Board and all methods were performed in accordance with the guidelines and regulations of this ethics board and the Hospital Ethics Committee agreed to the informed consent waiver.

## Results

### Patients

Patient characteristics are presented in Table 1. All these clinical features, expect pCR (pathologic complete response), which was calculated from pathologic nodal stage and pathologic tumor stage, were enrolled into our modeling to assess the dependence of the radiomics features.

**Table 1:** Patient Demographics and Clinical Characteristics.

Variable	No. of Patients	Local Control		Distant Control		Overall Survival		Disease-free Survival	
		5 years	P-value	5 years	P-value	5 years	P-value	5 years	P-value
Total No. of Patients	411	81.4	—	68.4	—	75.6	—	61.1	—
**Clinical diagnosis**									
Sex			0.272		0.830		0.188		0.657
Male	301	81.6		68.5		73.9		60.7	
Female	110	78.1		68.2		80.5		62.2	
Age, years			0.253		0.479		0.659		0.909
≤49	141	78.7		69.5		73.2		60.6	
50–59	124	82.0		69.0		76.7		63.2	
60–69	110	82.3		69.5		81.2		59.2	
≥70	36	87.1		60.8		68.6		60.8	
cT stage			<0.001^a^		0.684		0.002^a^		0.120
2	14	92.9		72.2		73.5		66.7	
3	301	84.2		69.4		79.0		63.4	
4	76	64.6		57.6		63.4		44.6	
missing	20								
cN stage			0.015^a^		0.013^a^		0.004^a^		0.002^a^
0	45	97.7		83.7		100.0		83.7	
1–2	326	78.1		66.1		72.4		57.3	
missing	40								
**Treatments**									
RT dose, Gy			0.193		0.042^a^		0.012^a^		0.011^a^
<50	85	75.1		56.8		61.4		47.6	
≥50	326	83.4		72.0		80.5		65.0	
Concurrent chemotherapy		0.246		0.909		0.603		0.623	
No	9	100.0		71.1		100.0		71.1	
Yes	402	81.0		68.3		75.1		60.9	
Surgical procedure			0.155		0.280		0.009^a^		0.152
LAR	178	81.7		65.8		77.1		59.5	
APR	216	82.9		71.7		76.9		63.6	
Hartmann	16	66.3		61.9		50.6		55.6	
missing	1								
Adjuvant chemotherapy		0.410		0.879		0.108		0.635	
No	39	81.4		70.1		71.6		56.5	
Yes	350	80.9		68.2		77.1		61.5	
missing	22								
**Pathology**									
pT stage			0.045^a^		0.004^a^		<0.001^a^		<0.001^a^
0	88	91.3		79.2		83.9		78.0	
1–2	122	82.9		74.5		89.0		65.9	
3	171	77.4		58.5		64.9		50.7	
4	30	64.3		70.4		54.3		47.3	
pN stage			0.002^a^		<0.001^a^		<0.001^a^		<0.001^a^
0	248	87.9		79.3		85.6		73.8	
1–2	163	69.3		51.0		59.2		40.9	
pCR			0.041^a^		0.062		0.205		0.007
No	323	79.2		0.658		73.0		56.8	
Yes	88	91.4		0.798		87.2		79.9	

NOTE. Significant differences between the stratified Kaplan-Meier curves are indicated by the P-value. Five years event values given as percentage.

Abbreviations: APR, abdominoperineal resection; cT stage, clinical tumor stage; cN, clinical nodal stage; LAR, low anterior resection; RT, radiotherapy; pT, pathologic tumor stage; pN, pathologic nodal stage. pCR, pathologic complete response.

^a^Significant overall difference: P < 0.05.

### Test-Retest and Contour-Recontour

**Figure 2:**
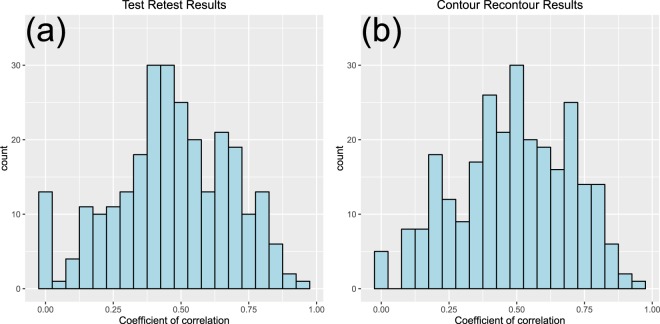
The distribution of the correlation of coefficient for the test-restest and contour-recontour studies.

Figure 2 presents the test-retest and contour-recontour results. According to our criteria, the test-retest study has 36 selected features, whereas the contour-recontour study has 41 selected features. Combining these two feature datasets, 21 features were selected. The details of the features selected are provided in the Supplementary Tables S2–S4. The final enrolled stable features include 2 types, including grey feature and texture features.

### NMF and cluster correlation results

**Figure 3:**
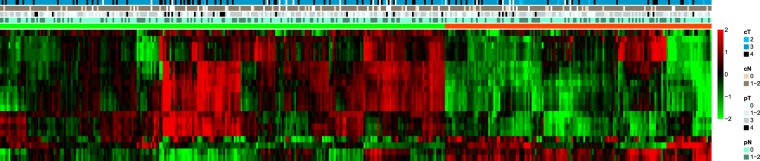
Non-negative matrix factorization cluster results. cT stage, clinical tumor stage; cN, clinical nodal stage; pT, pathologic tumor stage; pN, pathologic nodal stage.

**Figure 4:**
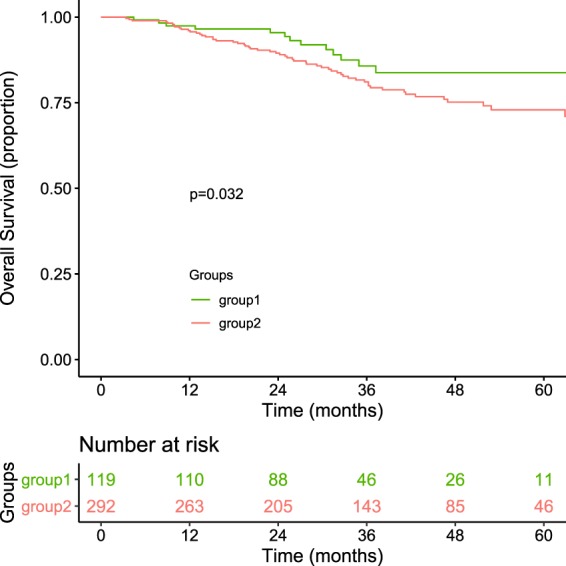
Overall survival curves for 2 groups.

The cluster results are presented in Fig. 3. Detailed information of the NMF can be found in Supplementary Figs S1–S2. Base on the consensus map and rank survey, rank 2 is the most appropriate for this study. Patients were split into two groups based on clusters. No clinical features were related to the cluster results. The results of chi-square test were presented in the Table 2. There was not correlation between patient characters and cluster results. The overall survival curve for the two groups are presented in Fig. 4. There was significant differences in overall survival (p = 0.032, Log-rank test) between two group. No differences for other outcomes, including distant control, local control and progression-free survival, were noted for these two groups. Detailed information is provided in the Supplementary Fig. S3.

**Table 2:** The results of chi-square test for this cluster with clinical features.

Patient Characters	p-value
clinical tumor stage	0.1265
clinical nodal stage	0.6763
pathologic nodal stage tumor	0.4655
pathologic nodal stage	0.9046
sex	0.3242
Age	0.4542
RT dose	0.8721
Surgery	0.8201

### Modeling performance

**Figure 5:**
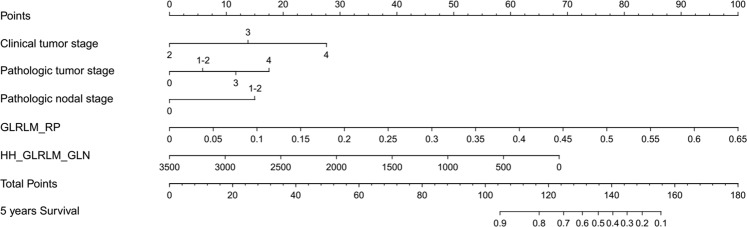
The nomogram of the overall survival prediction model.

The supervised model performance is presented in Table 3. The overall survival was improved by radiomics features from 0.67 to 0.73, suggesting that radiomics features are an independent feature of overall survival prediction. The paired t-test showed the c-index was significant difference between clinical model and mixed model (p = 0.044). For other endpoints, radiomics features do not provide additional information for distant control and progression-free survival prediction. Radiomics features provide information for local control prediction. Figure 5 presents the final model for the overall survival prediction. The details of the model parameters are provided in the Supplementary Table S5. The final enrolled radiomics factors in overall survival prediction included GLRLM_RP and HH_GLCM_GLN. GLRLM_RP is one of the Gray-level run lengths features, and RP (Run percentage) takes low values for smooth images. HH_GLCM_GLN is one the Gray-Level run length features with a transferred CT wavelet. GLN (Gray-level nonuniformity) takes small values when runs are uniformly distributed among the gray levels^27^. Basically, these two features are indexes of image homogeneity. Given that our patients were almost treated in one scheme, the final model does not reflect the influence of the treatment method.

**Table 3:** Model performance.

	Training			Testing		
	Radiomics	Clinical	Both	Radiomics	Clinical	Both
Local Control	0.643	0.692	0.733	0.563	0.637	0.651
	[0.622 0.665]	[0.680 0.705]	[0.719 0.748]	[0.465 0.660]	[0.536 0.737]	[0.554 0.747]
Distant Control	/	0.657	0.657	/	0.640	0.640
	/	[0.645 0.669]	[0.645 0.669]	/	[0.577 0.703]	[0.577 0.703]
Overall Survival	0.675	0.713	0.745	0.655	0.672	0.730
	[0.663 0.687]	[0.701 0.726]	[0.731 0.760]	[0.589 0.722]	[0.617 0.728]	[0.658 0.801]
Disease-free Survival	/	0.678	0.683	/	0.658	0.643
	/	[0.670 0.658]	[0.675 0.692]	/	[0.585 0.731]	[0.571 0.714]

Note, 95% confidence intervals are reported in the bracket. ‘/’ means no model can be established.

## Discussion

In this study, we investigated the feasibility of predicting outcomes for rectal cancer patients using radiomics features extracted from the planning CT. The results showed that radiomics features predict patients’ overall survival. As an independent prediction feature, radiomics features can combine with clinical features to provide better model performance for overall survival prediction.

No perfect method is available to predict patient cCR (clinical complete response) by traditional clinical evaluation. One study showed that only 21% of patients with pCR were correctly identified by preoperative digital rectal examination^28^. As a standard staging approach, restaging tumor after chemoradiotherapy with MRI is also not perfect^29^. However, the information we provided in this study cannot predict the tumor stage after chemoradiotherapy, where there is no relationship between radiomics features and pathologic tumor stage. Our model can predict patient overall survival using the treatment planning CT before chemoradiotherapy. From this point, this information can provide additional information to decide whether to implement the watch-and-wait strategy. For cCR patients with a low risk by our prediction, we may tend to choose the watch-and-wait strategy, which may benefit patient life quality. For high-risk patients, we may increase the treatment strength and not adopt a watch-and-wait strategy^30–34^.

The optimal follow-up recommendations after radical resection for colorectal cancer remain undefined. Few randomized controlled trials have correlated follow-up and cancer mortality. Identifying subgroups of patients at different risks can help identify the appropriate timing and imaging techniques in a more individualized fashion. The prediction of patient overall survival can benefit patient follow-up design.

Radiomics studies require a rigorous study design to ensure the reproducibility of the study^35^. In this study, we have taken many approaches to ensure the reproducibility of radiomics studies. First, we implemented test-retest and contour-recontour studies to remove unstable features. This process was indispensable for radiomics studies. As shown in our study, only 21 features were selected from 271 features. This selection not only increases the credibility of the entire study but also reduces the overfitting problem when modeling. Based on our experience, different sites exhibit different performances in feature reproducibility^36^. Second, we use two statistical methods, including the unsupervised method and supervised method to demonstrator the value of the radiomics features to the prognosis prediction. In addition, in supervised method. A 10-fold cross validation was implement to ensure that the model was not overfit. Our results also demonstrated that for overall survival prediction, the training c-index was similar to the testing c-index. For local control and disease-free survival, the training c-index was considerably increased than the testing c-index (0.733 to 0.651 and 0.657 to 0.640, respectively). This finding indicated that this model was overfit for local control and disease-free survival predictions. This finding may be explained because the events number was too small to generate a stable model. Third, we have carefully assessed the relationship between clinical features and radiomics features. The chi-square test showed that there is no relationship between clinical features and radiomics features. In the supervised method, we incorporated the clinical and radiomics features into model training and validation and treat them as clinical features. We do not create a ‘radiomics score’ before final modeling. During radiomics score generation, we believe that the outcome has been used for radiomics score generation. This feature will introduce bias upon final modeling.

The role of MR was increase in clinical, since this imaging modality already proven its validity in the characterization of tumor in more traditional fashion^37^. Mercury study have showed that high resolution magnetic resonance imaging could accurately predicts whether the surgical resection margins will be clear or affected by tumor^38^. The application of MR for radiomics has always been considered affected by many issues due to the intrinsic difficulty in generalizing the analysis of signal in MR images because of the problem of normalization and regularization of MR images^39^. CT images which have less parameters may more stable than MRI image. This is one of the reason we choose CT in this study. Meanwhile, the all treatment planning CT was acquired with similarly protocol due to radiotherapy requirement, such as the KV and mA value.

There was some limitation in this study. First, we do not have external validation in this study. Second, we have used a lot of method to remove influence of contouring and CT scanning. But, we believe these biases was still existing. A further study which include multi-institution may overcome these biases.

In this study, we used the entire volume of the tumor to calculate radiomics features. We believe that one of the advantage of radiomics is that it can capture information of the entire tumor not one slice of the tumor. Given intra-tumor heterogeneity^40^, the volume may capture more information than one slice. The medical image set used in this research involves the planning CTs of rectal cancer patients, and these data are routinely obtained for planning radiation therapy. As a result, this approach would also be less costly and time consuming than genetic or functional imaging techniques.

The primary results have present in 2017 ASTRO annual meeting^41^.

## Supplementary information

supplementary
