# Supplementary material for: Radiomics features on radiotherapy treatment planning CT can predict patient survival in locally advanced rectal cancer patients
Source: Sci Rep. 2019 Oct 25;9:15346. doi: 10.1038/s41598-019-51629-4 (PMC6814843; doi:10.1038/s41598-019-51629-4)
Supplement: Supplementary file 1 — supplementary [file 41598_2019_51629_MOESM1_ESM.docx]

Radiomics features on radiotherapy treatment planning CT can predict patient survival in locally advanced rectal cancer patients

Jiazhou Wang*^1^, Lijun Shen*^1^, Haoyu Zhong^2^, Zhen Zhou^3^, Panpan Hu^1^, Jiayu Gan^1^, Ruiyan Luo^1^, Weigang Hu^1^, Zhen Zhang^#1^

^1^ Department of Radiation Oncology, Fudan University Shanghai Cancer Center, Shanghai 200032, China and Department of Oncology, Shanghai Medical College, Fudan University, Shanghai 200032, China

^2^ Perelman Center for Advanced Medicine, Philadelphia, PA 19104, US

^3^ MAASTRO Clinic, Maastricht, Netherlands

Contents:

Supplementary Table S1. The feature of the radiomics features

Supplementary Table S2. Features selected by Test-Retest

Supplementary Table S3. Features selected by Contour-Recontour

Supplementary Table S4. Stable features (intersection of Test-Retest and Contour-Recontour)

Supplementary Figure S1. Consensusmap of NMF

Supplementary Figure S2. NMF rank survey

Supplementary Figure S3. Kaplan Meier curve for different group.

Supplementary Table S5. Model parameter.

Supplementary Table S1. The feature of the radiomics features

| ID | Feature name | Type |
| --- | --- | --- |
| 1 | volume | shape |
| 2 | area | shape |
| 3 | va_ratio | shape |
| 4 | scaled_mean | histogram |
| 5 | scaled_median | histogram |
| 6 | scaled_variance | histogram |
| 7 | scaled_skewness | histogram |
| 8 | scaled_kurtosis | histogram |
| 9 | scaled_entropy | histogram |
| 10 | scaled_min | histogram |
| 11 | scaled_max | histogram |
| 12 | scaled_std | histogram |
| 13 | scaled_range | histogram |
| 14 | absolute_mean | histogram |
| 15 | absolute_median | histogram |
| 16 | absolute_variance | histogram |
| 17 | absolute_skewness | histogram |
| 18 | absolute_kurtosis | histogram |
| 19 | absolute_min | histogram |
| 20 | absolute_max | histogram |
| 21 | absolute_std | histogram |
| 22 | absolute_range | histogram |
| 23 | GLCM_Contrast | GLCM |
| 24 | GLCM_Correlation | GLCM |
| 25 | GLCM_Energy | GLCM |
| 26 | GLCM_Homogeneity | GLCM |
| 27 | GLCM_Mean | GLCM |
| 28 | GLCM_Variance | GLCM |
| 29 | GLCM_Std | GLCM |
| 30 | GLCM_Dissimilarity | GLCM |
| 31 | GLCM_Entropy | GLCM |
| 32 | GLCM_Sum_average | GLCM |
| 33 | GLCM_Difference_average | GLCM |
| 34 | GLCM_Sum_variance | GLCM |
| 35 | GLCM_Difference_variance | GLCM |
| 36 | GLCM_Sum_Entropy | GLCM |
| 37 | GLCM_Difference_Entropy | GLCM |
| 38 | GLCM_Information_Measures_I | GLCM |
| 39 | GLCM_Information_Measures_II | GLCM |
| 40 | GLCM_Maximal_Correlation_Coefficient | GLCM |
| 41 | GLCM_Homogeneity_Original | GLCM |
| 42 | GLCM_Correlation_Original | GLCM |
| 43 | GLRLM_SRE | GLRLM |
| 44 | GLRLM_LRE | GLRLM |
| 45 | GLRLM_GLN | GLRLM |
| 46 | GLRLM_RLN | GLRLM |
| 47 | GLRLM_RP | GLRLM |
| 48 | GLRLM_LGRE | GLRLM |
| 49 | GLRLM_HGRE | GLRLM |
| 50 | GLRLM_SRLGE | GLRLM |
| 51 | GLRLM_SRHGE | GLRLM |
| 52 | GLRLM_LRLGE | GLRLM |
| 53 | GLRLM_LRHGE | GLRLM |
| 54 | LL_GLCM_Contrast | Wavelet |
| 55 | LL_GLCM_Correlation | Wavelet |
| 56 | LL_GLCM_Energy | Wavelet |
| 57 | LL_GLCM_Homogeneity | Wavelet |
| 58 | LL_GLCM_Mean | Wavelet |
| 59 | LL_GLCM_Variance | Wavelet |
| 60 | LL_GLCM_Std | Wavelet |
| 61 | LL_GLCM_Dissimilarity | Wavelet |
| 62 | LL_GLCM_Entropy | Wavelet |
| 63 | LL_GLCM_Sum_average | Wavelet |
| 64 | LL_GLCM_Difference_average | Wavelet |
| 65 | LL_GLCM_Sum_variance | Wavelet |
| 66 | LL_GLCM_Difference_variance | Wavelet |
| 67 | LL_GLCM_Sum_Entropy | Wavelet |
| 68 | LL_GLCM_Difference_Entropy | Wavelet |
| 69 | LL_GLCM_Information_Measures_I | Wavelet |
| 70 | LL_GLCM_Information_Measures_II | Wavelet |
| 71 | LL_GLCM_Maximal_Correlation_Coefficient | Wavelet |
| 72 | LL_GLCM_Homogeneity_Original | Wavelet |
| 73 | LL_GLCM_Correlation_Original | Wavelet |
| 74 | LH_GLCM_Contrast | Wavelet |
| 75 | LH_GLCM_Correlation | Wavelet |
| 76 | LH_GLCM_Energy | Wavelet |
| 77 | LH_GLCM_Homogeneity | Wavelet |
| 78 | LH_GLCM_Mean | Wavelet |
| 79 | LH_GLCM_Variance | Wavelet |
| 80 | LH_GLCM_Std | Wavelet |
| 81 | LH_GLCM_Dissimilarity | Wavelet |
| 82 | LH_GLCM_Entropy | Wavelet |
| 83 | LH_GLCM_Sum_average | Wavelet |
| 84 | LH_GLCM_Difference_average | Wavelet |
| 85 | LH_GLCM_Sum_variance | Wavelet |
| 86 | LH_GLCM_Difference_variance | Wavelet |
| 87 | LH_GLCM_Sum_Entropy | Wavelet |
| 88 | LH_GLCM_Difference_Entropy | Wavelet |
| 89 | LH_GLCM_Information_Measures_I | Wavelet |
| 90 | LH_GLCM_Information_Measures_II | Wavelet |
| 91 | LH_GLCM_Maximal_Correlation_Coefficient | Wavelet |
| 92 | LH_GLCM_Homogeneity_Original | Wavelet |
| 93 | LH_GLCM_Correlation_Original | Wavelet |
| 94 | HL_GLCM_Contrast | Wavelet |
| 95 | HL_GLCM_Correlation | Wavelet |
| 96 | HL_GLCM_Energy | Wavelet |
| 97 | HL_GLCM_Homogeneity | Wavelet |
| 98 | HL_GLCM_Mean | Wavelet |
| 99 | HL_GLCM_Variance | Wavelet |
| 100 | HL_GLCM_Std | Wavelet |
| 101 | HL_GLCM_Dissimilarity | Wavelet |
| 102 | HL_GLCM_Entropy | Wavelet |
| 103 | HL_GLCM_Sum_average | Wavelet |
| 104 | HL_GLCM_Difference_average | Wavelet |
| 105 | HL_GLCM_Sum_variance | Wavelet |
| 106 | HL_GLCM_Difference_variance | Wavelet |
| 107 | HL_GLCM_Sum_Entropy | Wavelet |
| 108 | HL_GLCM_Difference_Entropy | Wavelet |
| 109 | HL_GLCM_Information_Measures_I | Wavelet |
| 110 | HL_GLCM_Information_Measures_II | Wavelet |
| 111 | HL_GLCM_Maximal_Correlation_Coefficient | Wavelet |
| 112 | HL_GLCM_Homogeneity_Original | Wavelet |
| 113 | HL_GLCM_Correlation_Original | Wavelet |
| 114 | HH_GLCM_Contrast | Wavelet |
| 115 | HH_GLCM_Correlation | Wavelet |
| 116 | HH_GLCM_Energy | Wavelet |
| 117 | HH_GLCM_Homogeneity | Wavelet |
| 118 | HH_GLCM_Mean | Wavelet |
| 119 | HH_GLCM_Variance | Wavelet |
| 120 | HH_GLCM_Std | Wavelet |
| 121 | HH_GLCM_Dissimilarity | Wavelet |
| 122 | HH_GLCM_Entropy | Wavelet |
| 123 | HH_GLCM_Sum_average | Wavelet |
| 124 | HH_GLCM_Difference_average | Wavelet |
| 125 | HH_GLCM_Sum_variance | Wavelet |
| 126 | HH_GLCM_Difference_variance | Wavelet |
| 127 | HH_GLCM_Sum_Entropy | Wavelet |
| 128 | HH_GLCM_Difference_Entropy | Wavelet |
| 129 | HH_GLCM_Information_Measures_I | Wavelet |
| 130 | HH_GLCM_Information_Measures_II | Wavelet |
| 131 | HH_GLCM_Maximal_Correlation_Coefficient | Wavelet |
| 132 | HH_GLCM_Homogeneity_Original | Wavelet |
| 133 | HH_GLCM_Correlation_Original | Wavelet |
| 134 | LL_GLRLM_SRE | Wavelet |
| 135 | LL_GLRLM_LRE | Wavelet |
| 136 | LL_GLRLM_GLN | Wavelet |
| 137 | LL_GLRLM_RLN | Wavelet |
| 138 | LL_GLRLM_RP | Wavelet |
| 139 | LL_GLRLM_LGRE | Wavelet |
| 140 | LL_GLRLM_HGRE | Wavelet |
| 141 | LL_GLRLM_SRLGE | Wavelet |
| 142 | LL_GLRLM_SRHGE | Wavelet |
| 143 | LL_GLRLM_LRLGE | Wavelet |
| 144 | LL_GLRLM_LRHGE | Wavelet |
| 145 | LH_GLRLM_SRE | Wavelet |
| 146 | LH_GLRLM_LRE | Wavelet |
| 147 | LH_GLRLM_GLN | Wavelet |
| 148 | LH_GLRLM_RLN | Wavelet |
| 149 | LH_GLRLM_RP | Wavelet |
| 150 | LH_GLRLM_LGRE | Wavelet |
| 151 | LH_GLRLM_HGRE | Wavelet |
| 152 | LH_GLRLM_SRLGE | Wavelet |
| 153 | LH_GLRLM_SRHGE | Wavelet |
| 154 | LH_GLRLM_LRLGE | Wavelet |
| 155 | LH_GLRLM_LRHGE | Wavelet |
| 156 | HL_GLRLM_SRE | Wavelet |
| 157 | HL_GLRLM_LRE | Wavelet |
| 158 | HL_GLRLM_GLN | Wavelet |
| 159 | HL_GLRLM_RLN | Wavelet |
| 160 | HL_GLRLM_RP | Wavelet |
| 161 | HL_GLRLM_LGRE | Wavelet |
| 162 | HL_GLRLM_HGRE | Wavelet |
| 163 | HL_GLRLM_SRLGE | Wavelet |
| 164 | HL_GLRLM_SRHGE | Wavelet |
| 165 | HL_GLRLM_LRLGE | Wavelet |
| 166 | HL_GLRLM_LRHGE | Wavelet |
| 167 | HH_GLRLM_SRE | Wavelet |
| 168 | HH_GLRLM_LRE | Wavelet |
| 169 | HH_GLRLM_GLN | Wavelet |
| 170 | HH_GLRLM_RLN | Wavelet |
| 171 | HH_GLRLM_RP | Wavelet |
| 172 | HH_GLRLM_LGRE | Wavelet |
| 173 | HH_GLRLM_HGRE | Wavelet |
| 174 | HH_GLRLM_SRLGE | Wavelet |
| 175 | HH_GLRLM_SRHGE | Wavelet |
| 176 | HH_GLRLM_LRLGE | Wavelet |
| 177 | HH_GLRLM_LRHGE | Wavelet |
| 178 | LL_scaled_mean | Wavelet |
| 179 | LL_scaled_median | Wavelet |
| 180 | LL_scaled_variance | Wavelet |
| 181 | LL_scaled_skewness | Wavelet |
| 182 | LL_scaled_kurtosis | Wavelet |
| 183 | LL_scaled_entropy | Wavelet |
| 184 | LL_scaled_min | Wavelet |
| 185 | LL_scaled_max | Wavelet |
| 186 | LL_scaled_std | Wavelet |
| 187 | LL_scaled_range | Wavelet |
| 188 | LL_absolute_mean | Wavelet |
| 189 | LL_absolute_median | Wavelet |
| 190 | LL_absolute_variance | Wavelet |
| 191 | LL_absolute_skewness | Wavelet |
| 192 | LL_absolute_kurtosis | Wavelet |
| 193 | LL_absolute_min | Wavelet |
| 194 | LL_absolute_max | Wavelet |
| 195 | LL_absolute_std | Wavelet |
| 196 | LL_absolute_range | Wavelet |
| 197 | LH_scaled_mean | Wavelet |
| 198 | LH_scaled_median | Wavelet |
| 199 | LH_scaled_variance | Wavelet |
| 200 | LH_scaled_skewness | Wavelet |
| 201 | LH_scaled_kurtosis | Wavelet |
| 202 | LH_scaled_entropy | Wavelet |
| 203 | LH_scaled_min | Wavelet |
| 204 | LH_scaled_max | Wavelet |
| 205 | LH_scaled_std | Wavelet |
| 206 | LH_scaled_range | Wavelet |
| 207 | LH_absolute_mean | Wavelet |
| 208 | LH_absolute_median | Wavelet |
| 209 | LH_absolute_variance | Wavelet |
| 210 | LH_absolute_skewness | Wavelet |
| 211 | LH_absolute_kurtosis | Wavelet |
| 212 | LH_absolute_min | Wavelet |
| 213 | LH_absolute_max | Wavelet |
| 214 | LH_absolute_std | Wavelet |
| 215 | LH_absolute_range | Wavelet |
| 216 | HL_scaled_mean | Wavelet |
| 217 | HL_scaled_median | Wavelet |
| 218 | HL_scaled_variance | Wavelet |
| 219 | HL_scaled_skewness | Wavelet |
| 220 | HL_scaled_kurtosis | Wavelet |
| 221 | HL_scaled_entropy | Wavelet |
| 222 | HL_scaled_min | Wavelet |
| 223 | HL_scaled_max | Wavelet |
| 224 | HL_scaled_std | Wavelet |
| 225 | HL_scaled_range | Wavelet |
| 226 | HL_absolute_mean | Wavelet |
| 227 | HL_absolute_median | Wavelet |
| 228 | HL_absolute_variance | Wavelet |
| 229 | HL_absolute_skewness | Wavelet |
| 230 | HL_absolute_kurtosis | Wavelet |
| 231 | HL_absolute_min | Wavelet |
| 232 | HL_absolute_max | Wavelet |
| 233 | HL_absolute_std | Wavelet |
| 234 | HL_absolute_range | Wavelet |
| 235 | HH_scaled_mean | Wavelet |
| 236 | HH_scaled_median | Wavelet |
| 237 | HH_scaled_variance | Wavelet |
| 238 | HH_scaled_skewness | Wavelet |
| 239 | HH_scaled_kurtosis | Wavelet |
| 240 | HH_scaled_entropy | Wavelet |
| 241 | HH_scaled_min | Wavelet |
| 242 | HH_scaled_max | Wavelet |
| 243 | HH_scaled_std | Wavelet |
| 244 | HH_scaled_range | Wavelet |
| 245 | HH_absolute_mean | Wavelet |
| 246 | HH_absolute_median | Wavelet |
| 247 | HH_absolute_variance | Wavelet |
| 248 | HH_absolute_skewness | Wavelet |
| 249 | HH_absolute_kurtosis | Wavelet |
| 250 | HH_absolute_min | Wavelet |
| 251 | HH_absolute_max | Wavelet |
| 252 | HH_absolute_std | Wavelet |
| 253 | HH_absolute_range | Wavelet |
| 254 | Box-Dimension_1 | Fraction dimmission |
| 255 | Minkowsky-Dimension_1 | Fraction dimmission |
| 256 | Mass-Dimension_1 | Fraction dimmission |
| 257 | Box-Dimension_2 | Fraction dimmission |
| 258 | Minkowsky-Dimension_2 | Fraction dimmission |
| 259 | Mass-Dimension_2 | Fraction dimmission |
| 260 | Box-Dimension_3 | Fraction dimmission |
| 261 | Minkowsky-Dimension_3 | Fraction dimmission |
| 262 | Mass-Dimension_3 | Fraction dimmission |
| 263 | Box-Dimension_4 | Fraction dimmission |
| 264 | Minkowsky-Dimension_4 | Fraction dimmission |
| 265 | Mass-Dimension_4 | Fraction dimmission |
| 266 | Box-Dimension_5 | Fraction dimmission |
| 267 | Minkowsky-Dimension_5 | Fraction dimmission |
| 268 | Mass-Dimension_5 | Fraction dimmission |
| 269 | Box-Dimension_6 | Fraction dimmission |
| 270 | Minkowsky-Dimension_6 | Fraction dimmission |
| 271 | Mass-Dimension_6 | Fraction dimmission |

Supplementary Table S2. Features selected by Test-Retest

| ID | Feature name | ID | Feature name |
| --- | --- | --- | --- |
| 1 | absolute_median | 19 | LH_GLCM_Information_Measures_II |
| 2 | GLCM_Contrast | 20 | HL_GLCM_Information_Measures_II |
| 3 | GLCM_Information_Measures_II | 21 | HH_GLCM_Information_Measures_I |
| 4 | GLRLM_SRE | 22 | LL_GLRLM_RLN |
| 5 | GLRLM_LRE | 23 | LL_GLRLM_LRLGE |
| 6 | GLRLM_GLN | 24 | LH_GLRLM_RLN |
| 7 | GLRLM_RLN | 25 | LH_GLRLM_RP |
| 8 | GLRLM_RP | 26 | HL_GLRLM_RLN |
| 9 | GLRLM_LGRE | 27 | HL_GLRLM_RP |
| 10 | GLRLM_SRLGE | 28 | HH_GLRLM_GLN |
| 11 | LL_GLCM_Contrast | 29 | HH_GLRLM_RLN |
| 12 | LL_GLCM_Variance | 30 | HH_GLRLM_RP |
| 13 | LL_GLCM_Std | 31 | LL_absolute_mean |
| 14 | LL_GLCM_Dissimilarity | 32 | LL_absolute_median |
| 15 | LL_GLCM_Difference_average | 33 | HL_absolute_median |
| 16 | LL_GLCM_Difference_variance | 34 | Minkowsky.Dimension_1 |
| 17 | LL_GLCM_Difference_Entropy | 35 | Minkowsky.Dimension_2 |
| 18 | LH_GLCM_Information_Measures_I | 36 | Box.Dimension_5 |

Supplementary Table S3. Features selected by Contour-Recontour

| ID | Feature name | ID | Feature name |
| --- | --- | --- | --- |
| 1 | absolute_mean | 23 | LH_GLRLM_LRLGE |
| 2 | absolute_median | 24 | HL_GLRLM_GLN |
| 3 | GLRLM_SRE | 25 | HL_GLRLM_RLN |
| 4 | GLRLM_LRE | 26 | HL_GLRLM_RP |
| 5 | GLRLM_GLN | 27 | HL_GLRLM_LGRE |
| 6 | GLRLM_RLN | 28 | HL_GLRLM_HGRE |
| 7 | GLRLM_RP | 29 | HL_GLRLM_SRHGE |
| 8 | LL_GLCM_Contrast | 30 | HL_GLRLM_LRHGE |
| 9 | LL_GLCM_Difference_variance | 31 | HH_GLRLM_GLN |
| 10 | LH_GLCM_Correlation | 32 | HH_GLRLM_RLN |
| 11 | LH_GLCM_Correlation_Original | 33 | HH_GLRLM_RP |
| 12 | HL_GLCM_Information_Measures_I | 34 | HH_GLRLM_LRHGE |
| 13 | HH_GLCM_Correlation | 35 | LL_absolute_mean |
| 14 | HH_GLCM_Information_Measures_I | 36 | LL_absolute_median |
| 15 | HH_GLCM_Correlation_Original | 37 | LL_absolute_max |
| 16 | LL_GLRLM_GLN | 38 | LH_absolute_mean |
| 17 | LL_GLRLM_RLN | 39 | LH_absolute_median |
| 18 | LL_GLRLM_RP | 40 | HL_absolute_mean |
| 19 | LH_GLRLM_GLN | 41 | HL_absolute_median |
| 20 | LH_GLRLM_RLN | 42 | Box.Dimension_1 |
| 21 | LH_GLRLM_RP | 43 | Minkowsky.Dimension_2 |
| 22 | LH_GLRLM_SRHGE | 44 | Minkowsky.Dimension_3 |

Supplementary Table S4. Stable features (intersection of Test-Retest and Contour-Recontour)

| ID | Feature name | ID | Feature name |
| --- | --- | --- | --- |
| 1 | absolute_median | 12 | LH_GLRLM_RLN |
| 2 | GLRLM_SRE | 13 | LH_GLRLM_RP |
| 3 | GLRLM_LRE | 14 | HL_GLRLM_RLN |
| 4 | GLRLM_GLN | 15 | HL_GLRLM_RP |
| 5 | GLRLM_RLN | 16 | HH_GLRLM_GLN |
| 6 | GLRLM_RP | 17 | HH_GLRLM_RLN |
| 7 | LL_GLCM_Contrast | 18 | HH_GLRLM_RP |
| 8 | LL_GLCM_Difference_variance | 19 | LL_absolute_mean |
| 9 | HH_GLCM_Information_Measures_I | 20 | LL_absolute_median |
| 10 | LL_GLRLM_RLN | 21 | HL_absolute_median |
| 11 | Minkowsky.Dimension_2 |  |  |


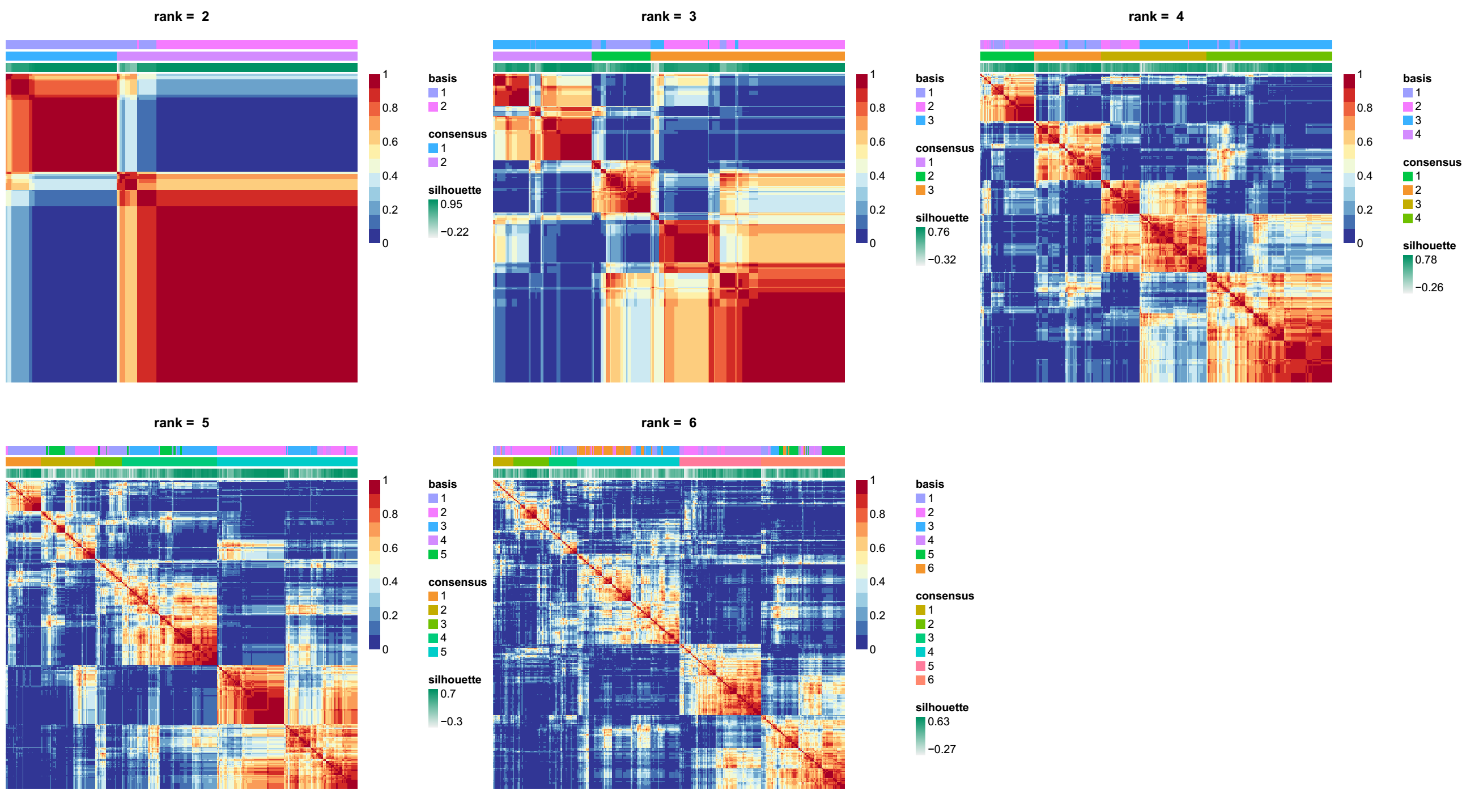


Supplementary Figure S1. Consensusmap of NMF


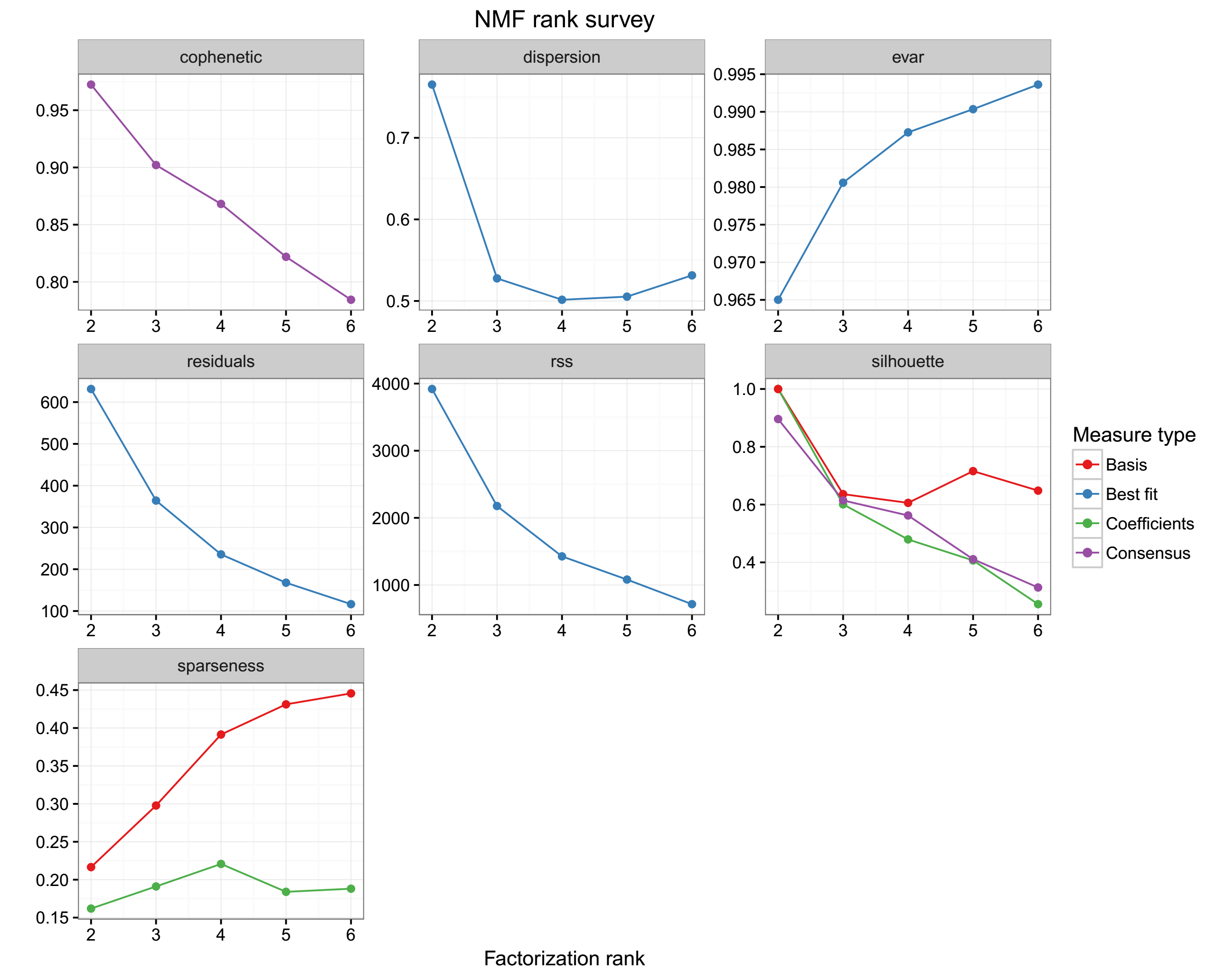


Supplementary Figure S2. NMF rank survey

| 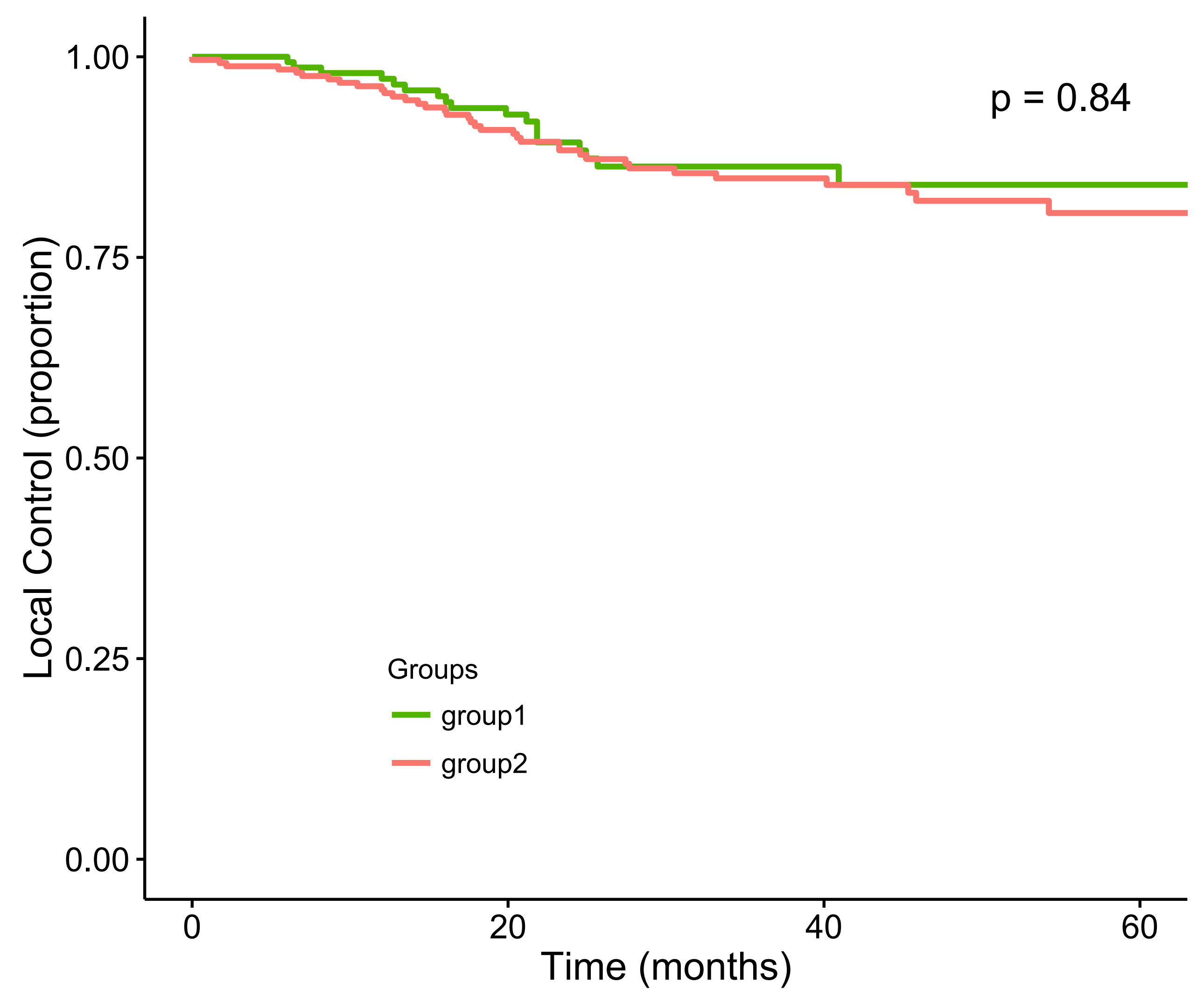 | 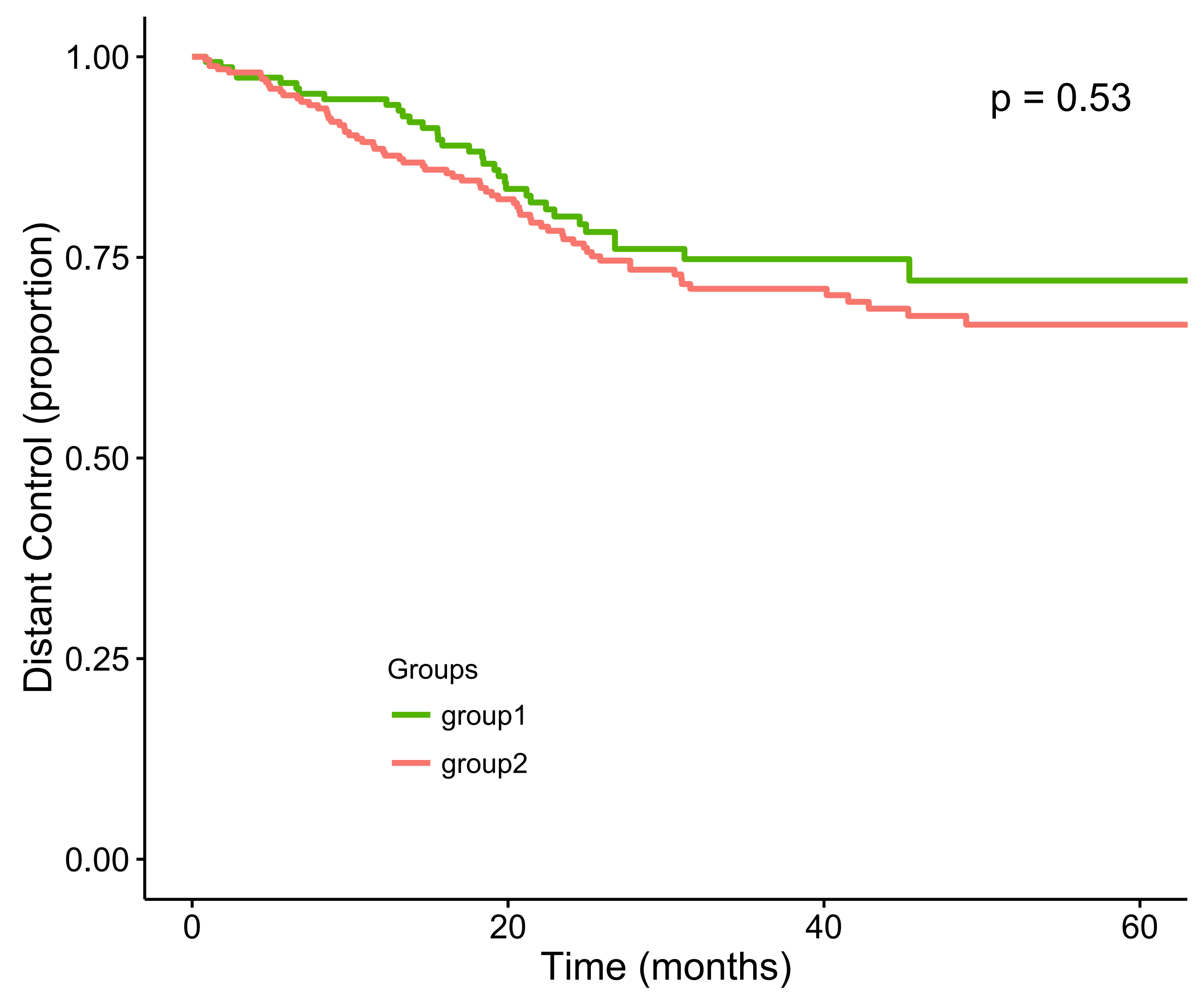 |
| --- | --- |
| 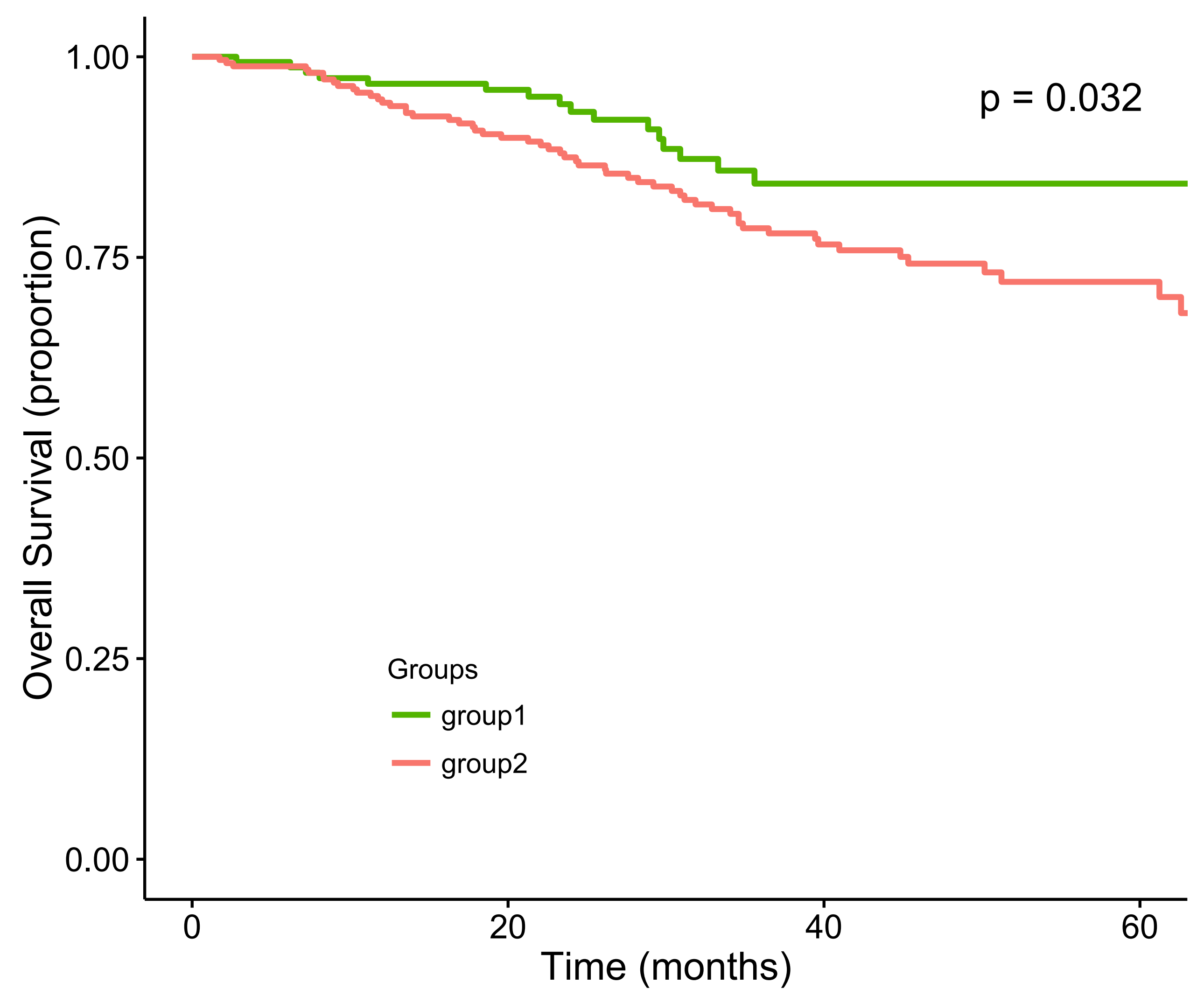 | 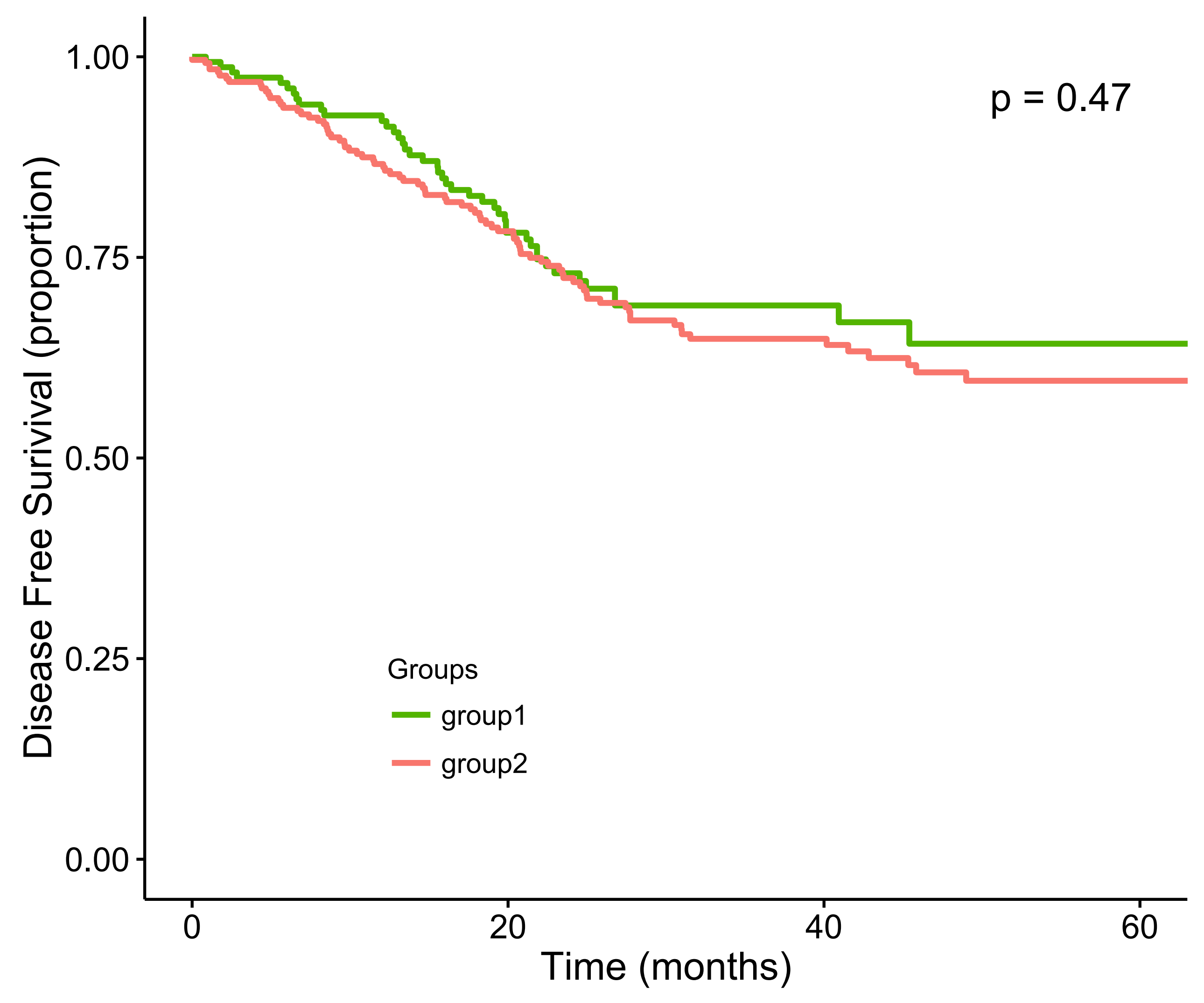 |

Supplementary Figure S3. Kaplan Meier curve for different group.

Supplementary Table S5. Model parameter.

|  | Coef | S.E. | Wald Z | Pr(>\|Z\|) |
| --- | --- | --- | --- | --- |
| Clinical tumor stage | 0.8361 | 0.2643 | 3.16 | 0.0016 |
| Pathologic tumor stage | 0.3533 | 0.1620 | 2.18 | 0.0292 |
| Pathologic nodal stage | 0.9071 | 0.2735 | 3.32 | 0.0009 |
| GLRKM_RP | 9.3157 | 2.1039 | 4.43 | <0.0001 |
| HH_GLCM_GLN | -0.0012 | 0.0004 | -2.65 | 0.0081 |
